# Use of immune repertoire sequencing to resolve discordant microscopic and immunochemical findings in a case of T cell-rich large B cell lymphoma in a young dog

**DOI:** 10.1186/s12917-021-02783-3

**Published:** 2021-02-18

**Authors:** Gary Kwok Cheong Lee, Dorothee Bienzle, Stefan Matthias Keller, Mei-Hua Hwang, Nikos Darzentas, Haiyang Chang, Emily Rätsep, Rebecca Egan, Janet Beeler-Marfisi

**Affiliations:** 1 Department of Pathobiology, University of Guelph, Guelph, Ontario Canada; 2 Department of Pathology, Microbiology & Immunology, University of California, Davis, CA USA; 3 Department of Internal Medicine II, University Hospital Schleswig-Holstein, Kiel, Germany; 4 Central European Institute of Technology, Masaryk University, Brno, Czech Republic

**Keywords:** Canine, Clonality, Flow cytometry, Immunohistochemistry, Liver, Neoplasia, Next-generation sequencing, PCR for antigen receptor gene rearrangements, TCRBL, Immunocytochemistry

## Abstract

**Background:**

Lymphocytic neoplasms with frequent reactive lymphocytes are uncommonly reported in dogs, and can pose a diagnostic challenge. Different diagnostic modalities such as cytology, flow cytometry, histopathology, immunohistochemistry, and clonality testing, are sometimes required for a diagnosis. This report illustrates the value of using a multi-modal diagnostic approach to decipher a complex lymphocytic tumor, and introduces immune repertoire sequencing as a diagnostic adjunct.

**Case presentation:**

A 10-month-old Great Dane was referred for marked ascites. Cytologic analysis of abdominal fluid and hepatic aspirates revealed a mixed lymphocyte population including numerous large lymphocytes, yielding a diagnosis of lymphoma. Flow cytometrically, abdominal fluid lymphocytes were highly positive for CD4, CD5, CD18, CD45, and MHC II, consistent with T cell lymphoma. Due to a rapidly deteriorating clinical condition, the dog was euthanized. Post mortem histologic evaluation showed effacement of the liver by aggregates of B cells surrounded by T cells, suggestive of hepatic T cell-rich large B cell lymphoma. Immune repertoire sequencing confirmed the presence of clonal B cells in the liver but not the abdominal fluid, whereas reactive T cells with shared, polyclonal immune repertoires were found in both locations.

**Conclusions:**

T cell-rich large B cell lymphoma is a rare neoplasm in dogs that may be challenging to diagnose and classify due to mixed lymphocyte populations. In this case, the results of histopathology, immunohistochemistry and immune repertoire sequencing were most consistent with a hepatic B cell neoplasm and reactive T cells exfoliating into the abdominal fluid. Immune repertoire sequencing was helpful in delineating neoplastic from reactive lymphocytes and characterizing repertoire overlap in both compartments. The potential pitfalls of equating atypical cytomorphology and monotypic marker expression in neoplasia are highlighted.

**Supplementary Information:**

The online version contains supplementary material available at 10.1186/s12917-021-02783-3.

## Background

Lymphoma encompasses diverse neoplastic entities characterized by clonal proliferations of lymphocytes. Similar to humans, multiple types of lymphoma have been described in the dog, but more than one type of lymphoma is rarely found concurrently within a patient [1–3]. Classification schemes for lymphoma are based on location, histomorphology and immunophenotype, and help predict progression and response to therapy [4]. The World Health Organization’s lymphoma classification scheme has been adapted for use in canine oncology, and one subtype of diffuse large B cell lymphoma in this classification scheme is T cell-rich large B cell lymphoma (TCRLBCL), which is characterized by neoplastic B cells within a more frequent population of small, non-neoplastic T cells [5]. Because they are a mixture of neoplastic and reactive lymphocytes, heterogeneous lymphocyte proliferations such as TCRLBCL can pose a diagnostic challenge. Reported cases of TCRLBCL in dogs are sparse, and include an ocular manifestation that progressed to a diffuse form in an 11-year-old dog [6] and another confined to the liver in a 7-month-old dog [7]. In both cases, aggregated or scattered large B cells intermixed with variable numbers of small T cells were reported histologically [6, 7]. There is also a report of an indolent hepatic lymphoma in an 11-year-old Golden Retriever that was comprised of clonal small B cells surrounding larger numbers of small to intermediate, mixed T cells, forming atypical follicular structures [8].

In specialty care facilities, a combination of diagnostic techniques including cytology, histology, immunohistochemistry (IHC), flow cytometry, and clonality testing is commonly used to diagnose and classify lymphoma. Cytology and histology both rely on cellular morphology to make a diagnosis of neoplasia, with histology having the advantage of architectural assessment [9]. Flow cytometry and IHC are adjuncts that allow immunophenotyping of lymphocyte populations, supplementing prior microscopic evaluation [4, 10]. Monotypic lymphocyte populations, often in combination with atypical cytomorphology, are supportive of a neoplastic process. However, in lymphomas with substantial reactive populations, it can be difficult distinguishing neoplastic from reactive lymphocytes. In ambiguous cases, clonality testing can be useful because it may distinguish between clonal and polyclonal lymphocyte populations by assessing the diversity of lymphocyte antigen receptor (LAR) genes [7, 11].

Traditional clonality testing assesses the diversity of LAR gene rearrangements by PCR amplification and size separation of amplicons by capillary electrophoresis [11–13]. This assay results in a semi-quantitative representation of lymphocyte clones stratified by size [11]. Due to recent advances in sequencing technology, high throughput sequencing followed by bioinformatical analysis can now be used to sequence PCR amplicons. This method is referred to as immune repertoire sequencing and offers higher sensitivity discrimination between lymphocyte populations since it distinguishes lymphocyte clones by the frequency of gene sequences rather than amplicon size [14, 15]. In addition to differentiating clonal from polyclonal lymphocyte proliferations, immune repertoire sequencing can be used to monitor minimal residual disease by tracking neoplastic lymphocyte clones over time, or to characterize reactive lymphocyte populations based on their LAR gene sequence [15].

This report describes the use of a multi-modal diagnostic approach, including immune repertoire sequencing, in deciphering a rare and complex lymphocytic neoplasm with conflicting diagnostic findings, in a young dog.

## Case presentation

A 10-month-old male intact Great Dane was referred to the Ontario Veterinary College Emergency Service for the sudden onset of ascites, and acute vomiting and diarrhea. The patient had a 2-week history of lethargy, hyporexia progressing to anorexia, and shifting hind limb pain that was unresponsive to 5 days of anti-inflammatory medication. Abnormalities on a complete blood cell count (CBC) included a mild mature neutrophilia (14.36 × 10^9^/L; reference interval [RI], 2.9–10.6 × 10^9^/L), mild left shift (band neutrophils 0.57 × 10^9^/L; RI, 0–0.3 × 10^9^/L) and mild monocytosis (1.89; RI, 0–1.1 × 10^9^/L), consistent with an inflammatory leukogram. Serum biochemical changes and urinalysis were interpreted to reflect dehydration, and effects of vomiting, diarrhea and ascites: hyperphosphatemia (2.89 mmol/L; RI 0.90–1.85 mmol/L), azotemia (urea 15.8 mmol/L; RI 3.5–9.0 mmol/L; creatinine 82 μmol/L; RI 20–150 μmol/L), urine specific gravity 1.041, hyponatremia (131 mmol/L; RI 140–154 mmol/L), hypochloremia (89 mmol/L; RI 104–119 mmol/L), panhypoproteinemia (albumin 26 g/L; RI 29–43 g/L, globulin 19 g/L; RI 21–42 g/L) and hypocholesterolemia (3.07 mmol/L; RI 3.60–10.20 mmol/L). Increased urea and normal creatinine values suggested gastrointestinal hemorrhage. There was evidence of a hepatopathy with hyperbilirubinemia (10 μmol/L; RI 0–4 μmol/L) and increased ALT activity (222 U/L; RI 19–107 U/L). Serologic test results for vector-borne infections (*Ehrlichia* spp., *Anaplasma* spp., *Borrelia burgdorferi*, *Dirofilaria immitis*) were negative (SNAP 4Dx Test, IDEXX, Lachine, QC).

**Fig. 1:**
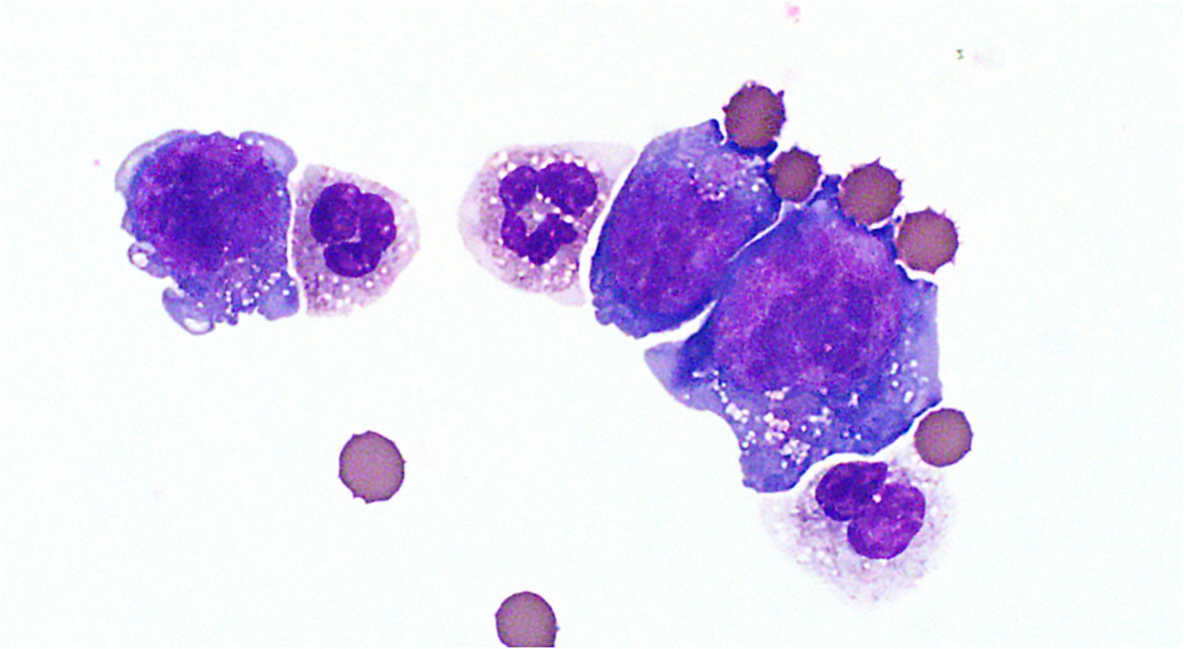
Hemorrhagic and lymphocytic effusion, abdominal fluid, dog. There were numerous large lymphocytes that often contained punctate cytoplasmic vacuolation and pink paranuclear granules. Nuclei were often irregular, contained fine chromatin and multiple, bizarrely shaped nucleoli. Wright stain, 1000x magnification

Four liters of turbid serosanguineous fluid were drained from the abdominal cavity. The cell concentration was 2.5 × 10^9^/L (RI 0–3 × 10^9^/L) and refractometric protein concentration was 42 g/L (RI 10–25 g/L). Both direct and cytocentrifuge preparations of the fluid contained a heterogeneous population of lymphocytes including small, intermediate and large round cells 10–30 μm in diameter. The large lymphocytes (Fig. 1) had a moderate amount of blue cytoplasm with frequent punctate vacuoles and fine pink paranuclear granules. The nucleus was located paracentrally, oval to irregularly shaped, with stippled chromatin and multiple prominent nucleoli. Frequent mitoses and rare binucleation were noted. Large lymphocytes (larger than neutrophils) made up 44% of nucleated cells, followed by 29% non-degenerate neutrophils, 21% small to intermediate lymphocytes, 4% macrophages, and 2% reactive mesothelial cells. The cytologic interpretation was a malignant round cell effusion, most likely lymphoma (Fig. 1).

**Fig. 2:**
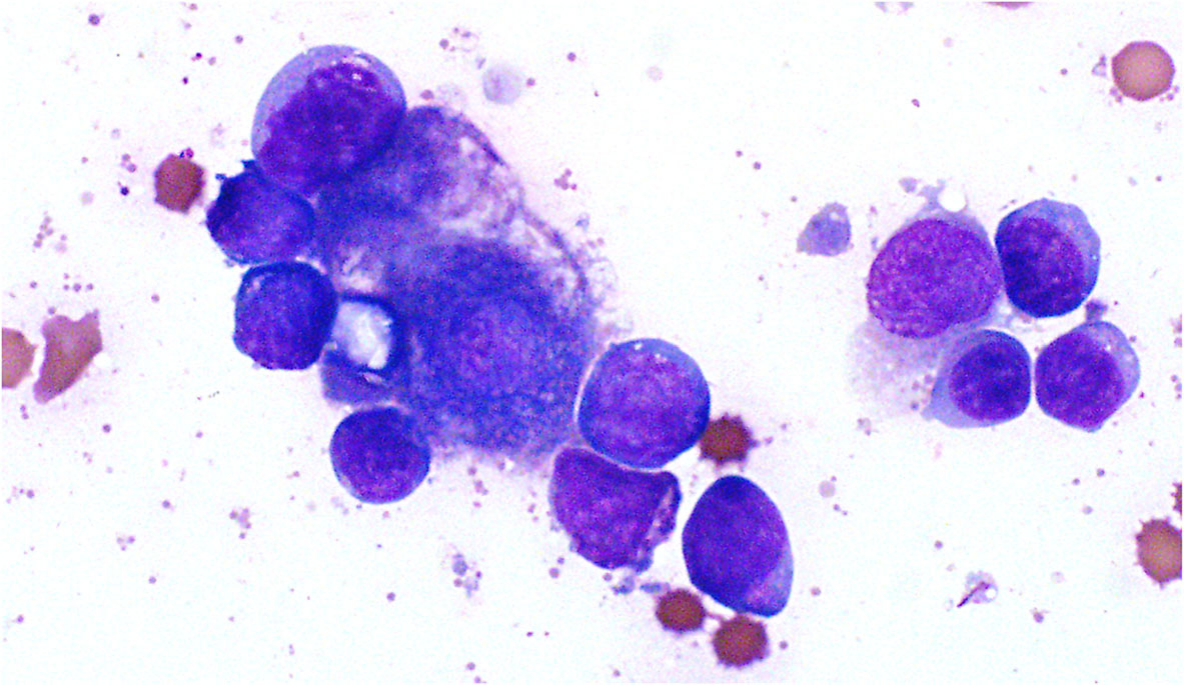
Lymphoma, liver, dog. Hepatocytes are surrounded by intermediate to large lymphocytes with similar cytologic features to those in abdominal fluid. Wright stain, 1000x magnification

An abdominal ultrasound and liver aspirate were performed the following day. The ultrasound revealed moderate amounts of peritoneal fluid and a mildly enlarged, diffusely hyperechoic liver with several ill-defined hypoechoic nodules up to 9.6 mm in diameter. Aspirate samples were highly cellular and, in addition to clusters of hepatocytes, contained numerous lymphocytes, heterogeneous in size, including large lymphocytes similar in appearance to those in the abdominal effusion (Fig. 2). Plasma cells and macrophages were noted in low numbers. The interpretation was round cell tumor, most likely lymphoma, involving the liver and exfoliating into the abdominal cavity. Based on the dog’s poor clinical condition and presumed guarded prognosis, euthanasia was elected.

**Fig. 3:**
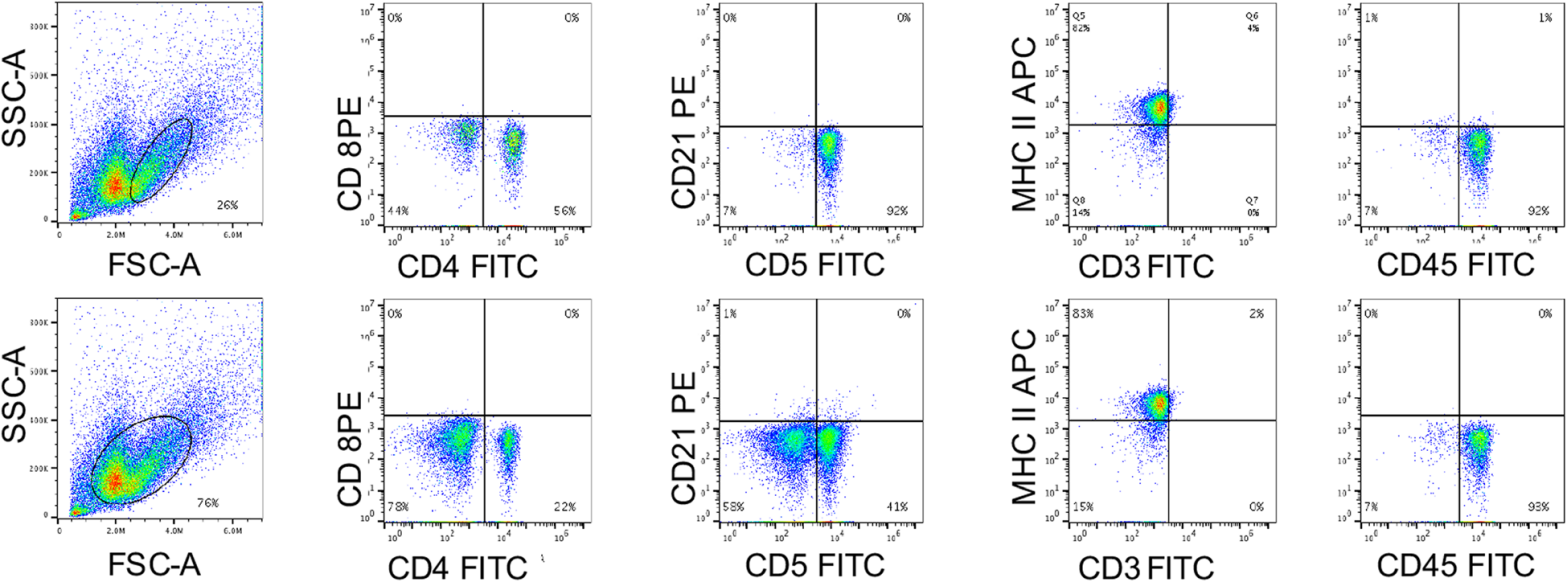
Flow cytometric analysis of abdominal effusion cells. Gating on cells with high forward scatter (FSC) and high side scatter (SSC) (top row) identifies cells that are nearly all positive for CD5, MHC II and CD45, and 56% that are positive for CD4. Note faint CD3 positivity on a few cells, and absence of CD8 and CD21 detection. Gating on cell populations with high and low FSC (bottom row) shows that almost all cells are MHC II and CD45 positive, confirming that they are lymphocytes. A proportion are also CD4 (22%) and CD5 (41%) positive. Detection of CD3 is faint, and CD8 or CD21 positive cells are rare

At postmortem examination, there were approximately four liters of serosanguineous fluid within the abdomen. The liver was enlarged, light brown, and contained numerous 1–8 mm diameter, white, flat to slightly raised foci. The spleen had rounded margins and two 10 mm diameter soft nodules that were mottled red and white on cut section. Multiple 1–8 mm diameter ulcers were present throughout approximately 10–15% of the pyloric mucosa and proximal duodenum. This finding supported the suspicion of gastrointestinal hemorrhage, which may have been associated with anti-inflammatory medication. Several mesenteric lymph nodes were moderately enlarged, dark red and firm. Abdominal fluid, collected at post mortem, was submitted for flow cytometric analysis [10]. There were small to medium cells with low side scatter, and forward scatter lower than that typical of neutrophils, as well as large cells with higher side scatter, and forward scatter similar to that of neutrophils (Fig. 3). Large cells with high side scatter were highly positive for CD4, CD5, CD18, CD45 and MHC II, faintly positive for CD3, and negative for CD21, CD22, CD14 and CD34 [16]. Inclusion of smaller cells in the analysis yielded similar results, but with a lower proportion of CD5-positive cells. These findings were interpreted to indicate an exfoliating T cell lymphoma.

**Fig. 4:**
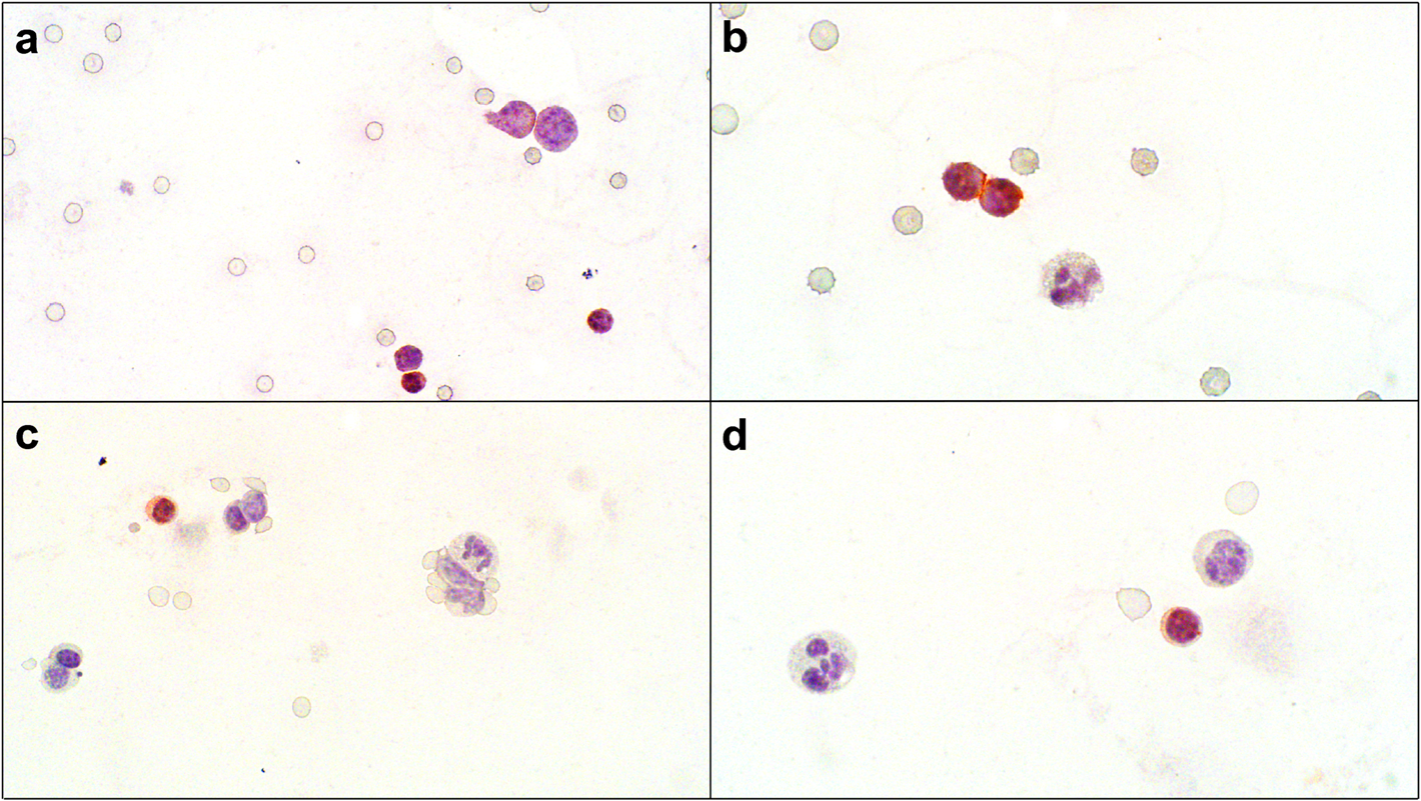
Immunocytochemistry (ICC) of abdominal effusion fluid collected at postmortem, dog. **a** Three small lymphocytes had strong cytoplasmic immunopositivity for CD3, while intermediate and large lymphocytes had faint cytoplasmic immunopositivity for CD3. The background contained numerous erythrocytes. ICC for CD3, 600x magnification. **b** Granulocytes were negative for CD3. ICC for CD3, 1000x magnification. **c** Rare small lymphocytes had cytoplasmic immunopositivity for CD20. The majority of the lymphocytes in the fluid were immunonegative for CD20. ICC for CD20, 600x magnification. **d** CD20 immunopositivity is apparent only in small lymphocytes. ICC for CD20, 1000x magnification

Sediment smears, prepared from fluid obtained post mortem, had similar cytologic findings to those of the antemortem fluid sample. Immunocytochemistry (ICC) for CD3 and CD20 was performed on abdominal fluid following a previously described protocol for Romanowski-stained slides [17]. Over 80% of lymphocytes had immunoreactivity for CD3. Small lymphocytes were strongly immunopositive for CD3 while medium and large lymphocytes were faintly immunopositive (Fig. 4a and b). Less than 10% of small lymphocytes had cytoplasmic immunoreactivity for CD20 (Figs. 4c and d). Occasional large lymphocytes were negative for both CD3 and CD20, precluding assignment of lineage to those cells. It was considered that some of these larger cells could be natural killer (NK) cells or undifferentiated leukocytes.

**Fig. 5:**
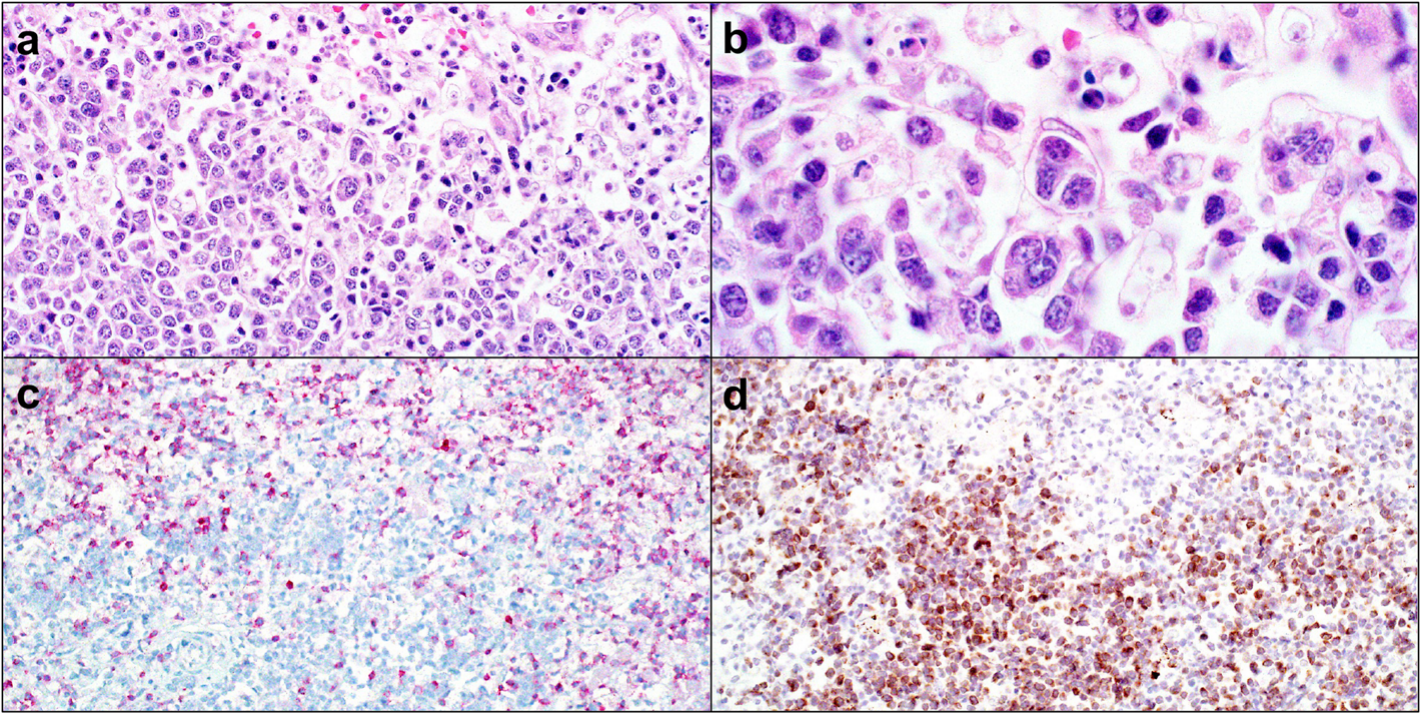
Lymphoma, liver, dog. **a** The hepatic parenchyma is effaced by aggregates of large lymphocytes surrounded by numerous small lymphocytes. HE, 400x magnification. **b** Higher magnification of field in Fig. 4a. Large lymphocytes have stippled chromatin whilst the smaller lymphocytes have dark, condensed chromatin. HE, 1000x magnification. **c** The small lymphocytes surrounding large aggregated lymphocytes had cytoplasmic immunopositivity (pink) for CD3, but large lymphocytes were negative. Immunohistochemistry (IHC) for CD3, 200x magnification. **d** The large aggregated lymphocytes had cytoplasmic immunopositivity for CD 79a (brown), but not the surrounding small lymphocytes. IHC for CD79a, 200x magnification

Formalin-fixed tissue sections of all major organs (liver, spleen, mesenteric lymph nodes, lung, kidneys, trachea, esophagus, peripheral nerves, tongue, skeletal muscle, bone marrow, thyroid glands, adrenal glands, diaphragm, stomach, duodenum, jejunum, ileum, cecum, colon, urinary bladder, synovium, testes, pituitary gland, brainstem, cerebral cortex) were routinely processed and stained with hematoxylin and eosin (HE). Histologically, approximately 80% of liver sections were effaced by aggregates of medium to large lymphocytes surrounded by sheets of small lymphocytes (Figs. 5a and b). Large lymphocytes had scant amphophilic cytoplasm, a round to irregular nucleus with coarsely stippled chromatin, and 3-fold anisokaryosis. There were up to seven mitotic figures per 400x magnification. The neoplasm was only identified in the liver. Both splenic nodules were consistent with nodular lymphoid hyperplasia and extramedullary hematopoiesis. The mesenteric lymph nodes were histologically unremarkable, and no cause for hind limb pain was elucidated.

**Fig. 6:**
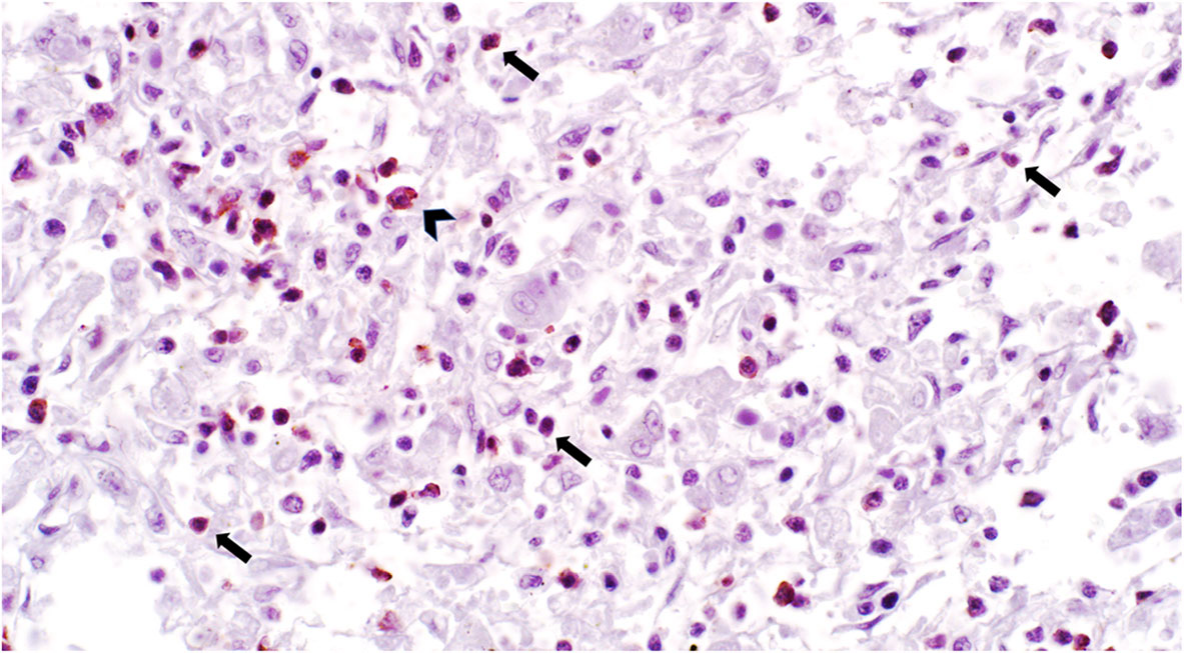
Lymphoma, liver, dog. The majority of small lymphocytes have cytoplasmic immunoreactivity to CD5 (arrows). Occasional intermediate-sized lymphocytes are also immunoreactive to CD5 (arrowhead). Immunohistochemistry for CD5, 400x magnification

To investigate the immunophenotype of hepatic lymphocytes, liver sections were evaluated by IHC for expression of CD3, CD5, CD20, and CD79a, as described previously [18, 19]. Small lymphocytes, comprising approximately 80% of all lymphocytes, had moderate to strong immunopositivity for CD3. The remaining 20% of lymphocytes were large and immunopositive for CD79a (Figs. 5c and d). Results of IHC with antibody to CD20 and CD79a were similar. Approximately 70% of the small lymphocytes and occasional intermediate-sized lymphocytes were immunopositive for CD5 (Fig. 6). These findings were suggestive of a TCRLBCL. Findings from histopathology, ICC and IHC were only in part consistent with those of flow cytometry.

**Fig. 7:**
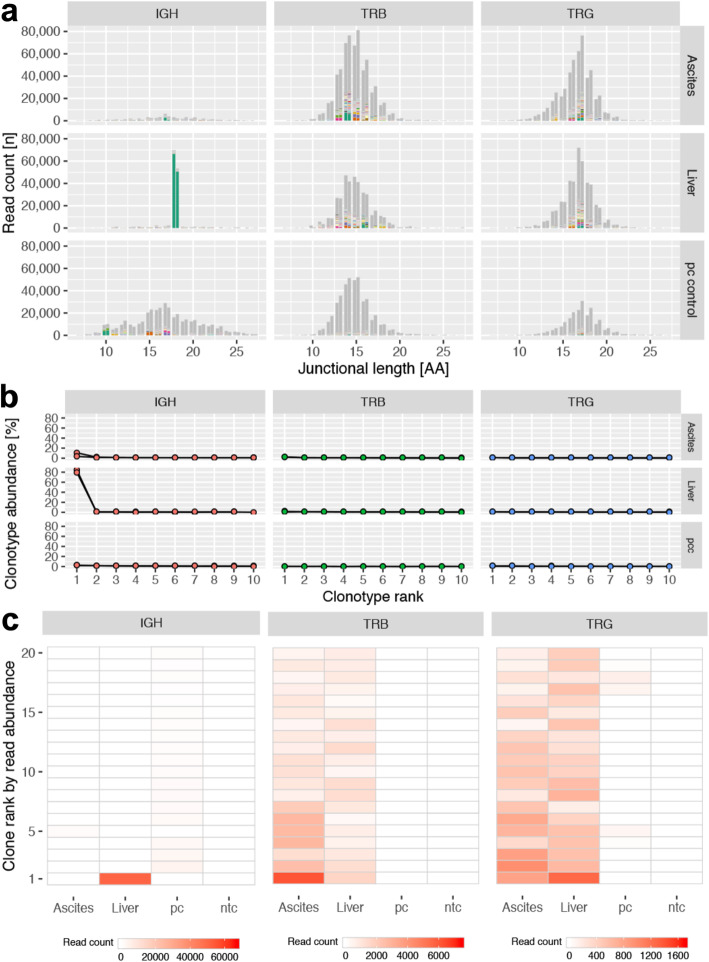
Immune repertoire analysis, liver and ascites, dog. **a** Bar plots depicting the lymphocyte antigen receptor gene diversity of lymphocyte infiltrates in ascites (top row) and liver (second row) of the immunoglobulin heavy chain locus (IGH) (left column), T cell receptor beta (TRB) (middle column) and T cell receptor gamma (TRG) (right column) loci as well as a lymph node from a dog without lymphoma (pc control). There is an unequivocal clonal IGH result for the liver sample; all other samples show polyclonal results for all loci consistent with reactive T cells. X-axis: junctional length in amino acids; y-axis: clonotype abundance in reads. Colored bar slices represent the 100 most abundant clonotypes per sample, grey bar slices are less abundant clonotypes. Double bars per junctional length indicate replicate samples. **b** Abundance of the 5 most frequent clonotypes, liver and ascites, dog. The most abundant IGH clonotype in the liver comprises greater than 80% of all reads and the line plot drops sharply after the most frequent clone; in contrast, all TRB and TRG clonotypes are below 3% forming a near horizontal line. Bottom row: polyclonal control (pcc). X-axis clonotype rank; y-axis clonotype abundance in percent of total reads per sample. **c** Heat map illustrating the overlap of the 20 most frequent IGH (left column), TRB (middle column) and TRG (right column) clonotypes in the liver and ascites of a dog with hepatic T cell-rich B cell lymphoma as well as a lymph node from a dog without lymphoma (pc control) and a negative control (nt control). Each column represents a sample and each row represents a clone. The color intensity of each tile corresponds to the read abundance supporting each clone; abundance is given as the mean of two replicates. The IGH repertoire is dominated by a single (neoplastic) clone with no discernible repertoire overlap between liver and ascites. In contrast, the TRB and TRG clonotypes are found in the liver and the ascites but not in the polyclonal control or the negative control

To resolve these conflicting findings, clonality testing and immune repertoire sequencing were performed on liver and ascites samples [1] [see Additional file 1 for materials and methods]. Briefly, DNA was extracted from cytological preparations of the hepatic aspirate and abdominal fluid. B and T cell LAR genes were amplified by multiplex PCR followed by both size separation of amplicons by capillary electrophoresis (clonality testing), and sequencing of amplicons by high-throughput sequencing (immune repertoire sequencing). These methods yielded concordant results with regard to the classification of samples as clonal or polyclonal [see Additional file 2 for gel electrophoresis results]. Details about the primer sets are provided in Additional file 3. Polyclonal results were obtained for the TRB and TRG loci of the liver and the ascites samples, suggesting that T cells in both compartments were reactive (Fig. 7a-c). Additionally, immune repertoire sequencing allowed quantification of clonotype abundance and determination of repertoire overlap between samples. Rank read counts of clonotypes are detailed in Additional file 4. The most abundant T cell clonotypes in the liver and abdominal effusion comprised 2.1 and 1.1% of the TRB and TRG loci, respectively (Fig. 7b). Additionally, the most abundant clones for both loci were present in abdominal fluid and liver samples, suggesting that hepatic T cells exfoliated into the abdominal effusion (Fig. 7c). In contrast, a clonal result was obtained for the IGH locus of the liver sample, implying that the hepatic B cells were neoplastic (Figs. 7a-c). The most abundant IGH clonotype constituted 82.1% of all reads and all other clonotypes constituted less than 0.5% of all reads (Fig. 7b). The ascites samples yielded polyclonal IGH results and fewer IGH reads than the liver sample, likely reflective of fewer B cells in the ascites fluid than the liver. The neoplastic B cell clone could be detected in the ascites sample, albeit at levels (0.009% of all IGH reads) that are too low to be distinguishable from incorrect sample assignment due to index hopping [20]. The final interpretation was hepatic large B cell lymphoma accompanied by reactive T cells, consistent with a TCRLBCL.

## Discussion and conclusions

This report describes the utility of immune repertoire sequencing in delineating the different lymphocyte populations in a case of canine TCRLBCL with confounding cytologic and flow cytometry findings. Canine TCRLBCL is a rare condition, with previous reports of TCRLBCL in dogs describing aggregates or scatterings of large B cells intermixed with variable numbers of small T cells [6, 7]. In our case, the B cells were aggregated and surrounded by, rather than admixed with, relatively large numbers of small T cells. These differing patterns raise the question of whether TCRLBCL in dogs could represent more than one disease entity. The low number of published cases of TCRLBCL in dogs precludes accurate prediction of disease outcome, but prognosis is suspected to be poor given the rapid disease progression despite chemotherapy in the reported cases [6, 7]. The severity of clinical illness in this case might also indicate greater similarity with human T cell/histiocyte-rich large B cell lymphoma (THRLBCL). However, the neoplastic B cells in THRLBCL do not form clusters or aggregates, whereas they did in the present case [21]. In humans, other B cell lymphomas that can present with a predominant, non-neoplastic T cell population are classical Hodgkin lymphoma, and the less common, nodular lymphocyte-predominant Hodgkin lymphoma [22]. Features associated with these neoplasms, including presence of Reed-Sternberg cells and lymphocyte-predominant or ‘popcorn’ cells, respectively, were not observed in this case.

In effusions, neoplastic lymphocytes may be challenging to differentiate from reactive lymphocytes by morphology, and the immunotypic properties of exfoliating lymphocytes in different inflammatory conditions remain to be determined [23]. In this case, the high proportion of large lymphocytes with atypical features including frequent mitoses, irregular nuclear shapes, and binucleation, suggested lymphoma. Flow cytometry is helpful in delineating lymphocyte populations based on size, complexity and immunophenotype, but does not indicate with certainty whether cells are neoplastic or reactive. Therefore, it has been suggested that flow cytometry be used to classify the type of lymphocytes in conjunction with cytologic or histopathologic assessment [24]. This case was particularly challenging since the cytologic findings were strongly suggestive of lymphoma, and the flow cytometric results were indicative of T cell predominance. Immunocytochemical staining for CD3 confirmed that the majority of the lymphocytes in the abdominal effusion were T cells, although on flow cytometry, CD3 was only detected faintly. The discrepancy between CD3 detection by flow cytometry and ICC may reflect exclusively membranous versus membranous and intracellular antigen detection in flow cytometry and ICC/IHC, respectively.

Immune repertoire sequencing in this case was pivotal for the diagnosis of hepatic B cell lymphoma, and for delineating the relationship of lymphocyte populations in liver and ascites fluid. In this study, the first rationale of immune repertoire sequencing was to substantiate the results of electrophoresis-based clonality testing, particularly to rule-out a neoplastic T cell clone obscured by a polyclonal background. Immune repertoire sequencing identified diverse TRB and TRG repertoires without dominant clones suggesting that T cells in the liver and ascites were a reactive population. While criteria for the interpretation of sequencing-based clonality testing results in veterinary diagnostic pathology are lacking, a false negative T cell clonality result was considered unlikely for the following reasons: 1) the two primer sets cover all pertinent variable and joining genes; 2) primer site mutation due to somatic hypermutation does not affect T cell receptor genes; 3) the canine TRG locus commonly carries more than one rearrangement providing additional redundancy for primer misses; 4) the independent assessment of two T cell loci further reduces the likelihood of a false negative result (concept of complementarity of targets); 5) robust amplification with both the TRB and TRG primer sets suggests that no major clone was missed; 6) the size distribution of T cell receptor genes follows a normal distribution; and 7) no dominant clonotypes were identified for either locus. It should be noted that the definition and significance of ‘dominant clonotypes’ for immune repertoire sequencing in dogs has not been established. However, the low abundance of the most dominant T cell clonotypes combined with the histological and IHC findings favored a reactive over a neoplastic T cell population. The variety of T cell sizes within the ascites, ranging from small to large, would also support a reactive population. Another possible explanation for the atypical cells observed on cytology in combination with the lack of a clonal result for the ascites sample would be an NK cell neoplasm. Natural killer cells do not rearrange antigen receptor genes and would hence go undetected by clonality testing or immune repertoire sequencing [25]. Given that some large cells appeared to be negative for both CD3 and CD20 on ICC, it is possible that there were some NK cells within the ascites fluid. However, the likelihood of a composite hepatic B cell lymphoma/abdominal NK cell tumor is extremely low and the robust T cell sequencing results from the ascites suggest that the majority of cells were indeed T cells. Reagents to unequivocally identify canine NK cells are unavailable.

The second rationale for using immune repertoire sequencing was to assess repertoire overlap between the ascites fluid and the liver. The results showed that T cells but not B cells in liver and ascites shared the same LAR gene sequences. This finding suggests differential homing or exfoliating properties for B and T cells in this case, i.e., T cells exfoliated much more readily into the effusion than B cells. This molecular evidence is supported by the microscopic findings of identifying primarily large B cells within the liver, but only rare small B cells within the ascites fluid on ICC. While the reason for this distribution is unclear, it illustrates that cavitary effusions might not be representative of the underlying tissue-based disease process.

In summary, this case report describes a rare type of B cell lymphoma in a young dog, which represented a diagnostic challenge due to the predominance of T cells with atypical cytomorphology in abdominal fluid. This case should alert diagnosticians to the value of a multi-modal diagnostic approach in unusual lymphoproliferative conditions, and illustrates the power of immune repertoire sequencing to discern clonal relationships and lymphocyte trafficking across anatomic sites.

## Supplementary Information

**Additional file 1.** Materials and Methods.

**Additional file 2: Figure.** PCR for antigen receptor rearrangement, liver and ascites, dog.

**Additional file 3: Table.** TCR primer sequences.

**Additional file 4: Table.** Rank read count of clones.

## Data Availability

All data generated or analyzed during this study are included in this published article, and Additional files 2, 3 and 4.

## Abbreviations

ALT: Alanine aminotransferase
CBC: Complete blood count
HE: Hematoxylin and eosin
ICC: Immunocytochemistry
IGH: Immunoglobulin heavy chain
IHC: Immunohistochemistry
LAR: Lymphocyte antigen receptor
PCR: Polymerase chain reaction
TCRLBCL: T cell-rich large B cell lymphoma
THRLBCL: T cell/histiocyte-rich large B cell lymphoma
TRB: T cell receptor beta
TRG: T cell receptor gamma
