# Supplementary material for: Use of immune repertoire sequencing to resolve discordant microscopic and immunochemical findings in a case of T cell-rich large B cell lymphoma in a young dog
Source: BMC Vet Res. 2021 Feb 18;17:85. doi: 10.1186/s12917-021-02783-3 (PMC7890612; doi:10.1186/s12917-021-02783-3)
Supplement: Supplementary file 1 — Additional file 1. Materials and Methods. [file 12917_2021_2783_MOESM1_ESM.docx]

**Additional file 1: Materials and Methods**

The general workflow outlined below has been described previously [1].

**DNA extraction and PCR**

DNA was extracted from scrapings of hepatic aspirate and abdominal fluid preparations using the QIAamp DNA Micro Kit (Qiagen, Valencia, CA, USA). DNA extracted from a frozen lymph node of a healthy dog was used as the polyclonal control and molecular biology grade water was used as the no template control. The extracted DNA was quantified by spectrophotometry (Nanodrop; ThermoFisher, Mississauga, ON, Canada) and normalized to 10 ng/μL. The rearranged canine immunoglobulin heavy-chain (IGH) genes, the canine T cell receptor beta (TRB) genes and the T cell receptor gamma (TRG) genes were amplified by multiplex PCR using forward primers targeting variable genes and reverse primers targeting joining genes. The IGH primer set has been described previously [2]. The TRB and TRG primer sequences are given in Additional file 4. The TRG primer set covers all functional, ORF, and pseudogene V and J germline genes. The TRB primer set covers all functional and ORF V and J germline genes and broad coverage of all rearranged genes was verified by 5’RACE transcriptome analysis. Primers had a maximum of 2 mismatches with the target sequence. PCR was performed using the New England Biolabs’ Q5® High-Fidelity Polymerase in replicate according to the manufacturer’s instructions and was visualized by capillary electrophoresis (High Resolution Cartridge, OL 500; Qiagen). The workflow up to this point resembled traditional clonality testing. After successful visualization, PCR products were pooled by replicate as follows (rep=replicate):

Pool rep1: IGH-rep1 + TRB-rep1 + TRG-rep1

Pool rep2: IGH-rep2 + TRB-rep2 + TRG-rep2

**Library preparation, sequencing and bioinformatics analysis**

Products were cleaned-up through a modified solid-phase reversible immobilization bead protocol using Mag-Bind RXNpure Plus beads (Omega Bio-Tek, Norcross, GA, USA). End prep and adapter ligation were performed using the NEBNext Ultra II DNA Library Prep Kit for Illumina (E7645; New England Biolabs, Ipswich, MA, USA) according to the manufacturer’s instructions. Samples were then fluorometrically quantified (Qubit dsDNA HS Assay Kit and Qubit 2.0 Fluorometer; Invitrogen, Carlsbad, CA, USA) and normalized to 4 nM prior to final pooling.

The library was sequenced on an Illumina Miseq instrument using the v2 PE300 reagent kit (Illumina, San Diego, CA, USA). The library was loaded at a concentration of 10 pM with 10% 12.5 pM PhiX by volume. Sequence demultiplexing and retrieval were performed using Illumina’s BaseSpace. All raw sequencing data are available through the Sequence Read Archive BioProject PRJNA579591.

Quality trimming and filtering were performed using the Trimmomatic software (PHRED 30, length 50 bp) [3]. Antigen receptor gene-specific analyses were performed using the ARResT/Interrogate software and custom R scripts [4]. A clone was defined as a unique junctional (CDR3 plus anchors) lymphocyte antigen receptor amino acid sequence. Variable and joining gene usage were identified as part of the workflow but were not reported. Given the lack of diagnostic criteria for sequencing-based clonality testing in veterinary medicine, electrophoresis and sequencing-based results were classified based on their junctional length distribution into: clonal – one or multiple reproducible peaks/bands with minimal amounts of other products; polyclonal – a normally distributed curve/a smear, as noted in the histogram view or gel electrophoresis view [5].

**References**

1. Matsuyama A, Bienzle D, Richardson D, Deravi N, Hwang MH, Darzentas N, et al. Composite lymphoma of concurrent T zone lymphoma and large cell B cell lymphoma in a dog. BMC Vet Res. 2019;15(1):413.

2. Hwang MH, Darzentas N, Bienzle D, Moore PF, Guscetti F, Morrison J, et al. A review of canine B cell clonality assays and primer set optimization using large-scale repertoire data. Vet Immunol Immunopathol. 2019;209:45-52.

3. Bolger AM, Lohse M, Usadel B. Trimmomatic: a flexible trimmer for Illumina sequence data. Bioinformatics. 2014;30(15):2114-20.

4. Bystry V, Reigl T, Krejci A, Demko M, Hanakova B, Grioni A, et al. ARResT/Interrogate: an interactive immunoprofiler for IG/TR NGS data. Bioinformatics. 2017;33(3):435-7.

5. Keller SM, Vernau W, Moore PF. Clonality Testing in Veterinary Medicine: A Review With Diagnostic Guidelines. Vet Pathol. 2016;53(4):711-25.
