# Supplementary material for: Use of immune repertoire sequencing to resolve discordant microscopic and immunochemical findings in a case of T cell-rich large B cell lymphoma in a young dog
Source: BMC Vet Res. 2021 Feb 18;17:85. doi: 10.1186/s12917-021-02783-3 (PMC7890612; doi:10.1186/s12917-021-02783-3)
Supplement: Supplementary file 2 — Additional file 2: Figure. PCR for antigen receptor rearrangement, liver and ascites, dog. [file 12917_2021_2783_MOESM2_ESM.zip › Additional file 2 legend_Dec_20.docx]

**Additional file 2 legend**

**Additional file 2.** PCR for antigen receptor rearrangement, liver and ascites, dog. A discrete clonal product for the immunoglobulin heavy chain locus (IGH) is noted at approximately 160 kb in the lane representing the liver sample. Discrete amplicons of the IGH locus were identified in neither the ascites sample nor the no template control (ntc). Polyclonal amplification products were noted in the ascites and liver samples for both the T cell receptor beta (TRB) and T cell receptor gamma (TRG) loci, and in all polyclonal controls (ptc). All amplifications used high-fidelity, low-yield polymerase. Peak size is in base pairs (bp). Red arrows: alignment markers (15 and 600 bp); green arrows: presumed primer dimer; black boxes: regions of interest.
