# Supplementary figures and images for: Use of immune repertoire sequencing to resolve discordant microscopic and immunochemical findings in a case of T cell-rich large B cell lymphoma in a young dog
Source: BMC Vet Res. 2021 Feb 18;17:85. doi: 10.1186/s12917-021-02783-3 (PMC7890612; doi:10.1186/s12917-021-02783-3)

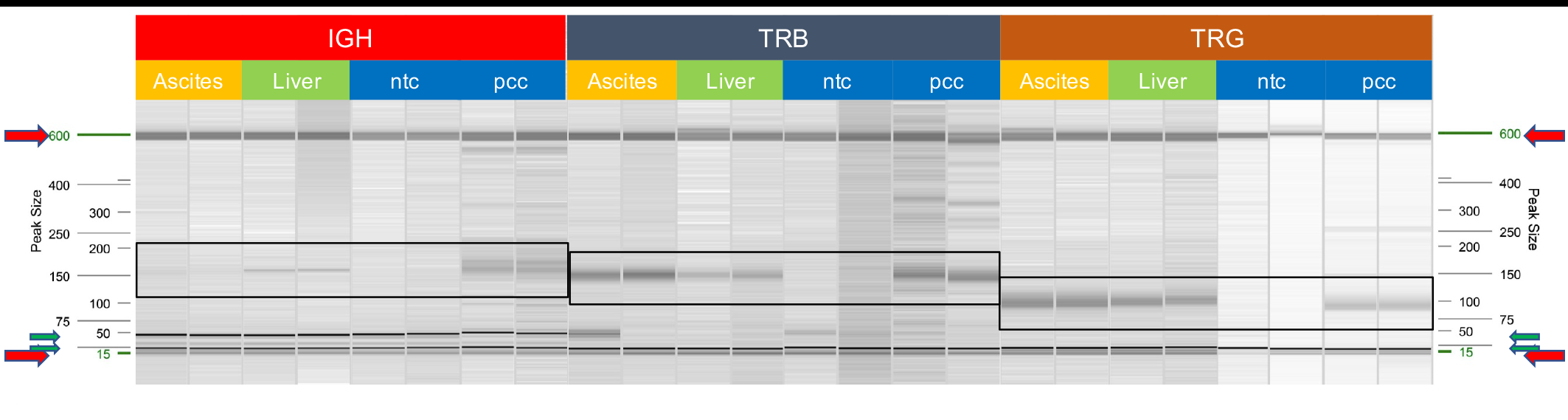

Supplement: Supplementary file 2 — Additional file 2: Figure. PCR for antigen receptor rearrangement, liver and ascites, dog. [file 12917_2021_2783_MOESM2_ESM.zip › Additional file 2_Dec_20.tif]
